# Supplementary material for: Accounting for overdispersion in skilled antenatal care: Identifying determinants using Bangladesh Demographic and Health Survey 2022 data
Source: PLoS One. 2026 Apr 8;21(4):e0346258. doi: 10.1371/journal.pone.0346258 (PMC13061173; doi:10.1371/journal.pone.0346258)
Supplement: Attachment — Submitted filename: PONE-D-25-34622.pdf [file pone.0346258.s003.pdf]

PLOS ONE

Accounting for overdispersion in skilled antenatal care: Identifying determinants using Bangladesh Demographic and Health Survey 2022 data

--Manuscript Draft--

**Manuscript Number:**	PONE-D-25-34622
**Article Type:**	Research Article
**Full Title:**	Accounting for overdispersion in skilled antenatal care: Identifying determinants using Bangladesh Demographic and Health Survey 2022 data
**Short Title:**	Accounting for overdispersion in skilled antenatal care: Identifying determinants using BDHS 2022 data
**Corresponding Author:**	Farhia Azrin<br>University of Dhaka<br>Dhaka, BANGLADESH
**Keywords:**	Maternal mortality; skilled antenatal care; dispersion test; generalized poisson regression
**Abstract:**	One of the major components required to ensure safe motherhood is taking a sufficient number of skilled antenatal care (SANC) visits by women during pregnancy. Various social, demographic, and economic factors have a strong influence on it. According to the World Health Organization (WHO), four or more antenatal care (ANC) visits are required for uncomplicated pregnancies and delivery of a healthy baby. SANC serves this purpose efficiently. As a result, modeling the number of SANC visits and identifying its determinants is crucial for the Bangladesh government in adopting appropriate policies and programs. In this study, we used score tests to assess dispersion in the count data and selected the Generalized Poisson Regression Model (GPRM) based on the lowest AIC (15,839.2) and BIC (16,001.7) values. Analysis of BDHS 2022 data (n = 3,839) revealed that only 38.6% of women received the recommended four or more SANC visits, with an overall mean of 3.06 visits. Key predictors of higher SANC utilization included urban residence (IRR = 1.09, p < 0.01), higher women's education (IRR = 1.57, p < 0.001), exposure to media (IRR = 1.16, p < 0.001), and being in the richest wealth quintile (IRR = 1.22, p < 0.001). In contrast, women in the Sylhet region (IRR = 0.89, p = 0.009) and those with higher birth order had lower SANC visit rates.
**Order of Authors:**	Md. Muddasir Hossain Akib, MS
	Farhia Azrin, MS
	Bikash Pal, MS
	Md. Mustain Billah, MS
**Additional Information:**	
**Question**	**Response**
**Financial Disclosure**<br><br>Enter a financial disclosure statement that describes the sources of funding for the work included in this submission. Review the submission guidelines for detailed requirements. View published research articles from *PLOS ONE* for specific examples.<br><br>This statement is required for submission and **will appear in the published article** if	The author(s) received no specific funding for this work.

Powered by Editorial Manager® and ProduXion Manager® from Aries Systems Corporation

the submission is accepted. Please make sure it is accepted.<br><br>**Funded studies**<br>Enter a statement with the following details:<br>• Initials of the authors who received each award<br>• Grant numbers awarded to each author<br>• The full name of each funder<br>• URL of each funder website<br>• Did the sponsors or funders play any role in the study design, data collection and analysis, decision to publish, or preparation of the manuscript?<br><br>Did you receive funding for this work?	
**Competing Interests**<br><br>Use the instructions below to enter a competing interest statement for this submission. On behalf of all authors, disclose any competing interests that could be perceived to bias this work—acknowledging all financial support and any other relevant financial or non-financial competing interests.<br><br>This statement is required for submission and **will appear in the published article** if the submission is accepted. Please make sure it is accurate and that any funding sources listed in your Funding Information later in the submission form are also declared in your Financial Disclosure statement.<br><br>View published research articles from *PLOS ONE* for specific examples.	The authors have declared that no competing interests exist.

**NO authors have competing interests**<br><br>Enter: *The authors have declared that no competing interests exist.*<br><br>**Authors with competing interests**<br><br>Enter competing interest details beginning with this statement:<br><br>*I have read the journal's policy and the authors of this manuscript have the following competing interests: [insert competing interests here]*<br><br>* typeset	
**Ethics Statement**<br><br>Enter an ethics statement for this submission. This statement is required if the study involved:<br><br>• Human participants<br>• Human specimens or tissue<br>• Vertebrate animals or cephalopods<br>• Vertebrate embryos or tissues<br>• Field research<br><br>Write "N/A" if the submission does not require an ethics statement.<br><br>General guidance is provided below. Consult the submission guidelines for detailed instructions. **Make sure that all information entered here is included in the Methods section of the manuscript.**	N/A

Powered by Editorial Manager® and ProduXion Manager® from Aries Systems Corporation

**Format for specific study types**<br><br>**Human Subject Research (involving human participants and/or tissue)**<br>• Give the name of the institutional review board or ethics committee that approved the study<br>• Include the approval number and/or a statement indicating approval of this research<br>• Indicate the form of consent obtained (written/oral) or the reason that consent was not obtained (e.g. the data were analyzed anonymously)<br><br>**Animal Research (involving vertebrate animals, embryos or tissues)**<br>• Provide the name of the Institutional Animal Care and Use Committee (IACUC) or other relevant ethics board that reviewed the study protocol, and indicate whether they approved this research or granted a formal waiver of ethical approval<br>• Include an approval number if one was obtained<br>• If the study involved *non-human primates*, add *additional details* about animal welfare and steps taken to ameliorate suffering<br>• If anesthesia, euthanasia, or any kind of animal sacrifice is part of the study, include briefly which substances and/or methods were applied<br><br>**Field Research**<br><br>Include the following details if this study involves the collection of plant, animal, or other materials from a natural setting:<br>• Field permit number<br>• Name of the institution or relevant body that granted permission	
**Data Availability**<br><br>Authors are required to make all data underlying the findings described fully available, without restriction, and from the time of publication. PLOS allows rare exceptions to address legal and ethical concerns. See the PLOS Data Policy and FAQ for detailed information.	Yes - all data are fully available without restriction

A Data Availability Statement describing where the data can be found is required at submission. Your answers to this question constitute the Data Availability Statement and **will be published in the article**, if accepted.<br><br>**Important:** Stating 'data available on request from the author' is not sufficient. If your data are only available upon request, select 'No' for the first question and explain your exceptional situation in the text box.<br><br>Do the authors confirm that all data underlying the findings described in their manuscript are fully available without restriction?	
**Describe where the data may be found in full sentences. If you are copying our sample text, replace any instances of XXX with the appropriate details.**<br><br>• If the data are **held or will be held in a public repository**, include URLs, accession numbers or DOIs. If this information will only be available after acceptance, indicate this by ticking the box below. For example: *All XXX files are available from the XXX database (accession number(s) XXX, XXX.).*<br>• If the data are all contained **within the manuscript and/or Supporting Information files**, enter the following: *All relevant data are within the manuscript and its Supporting Information files.*<br>• If neither of these applies but you are able to provide **details of access elsewhere**, with or without limitations, please do so. For example:<br><br>*Data cannot be shared publicly because of [XXX]. Data are available from the XXX Institutional Data Access / Ethics Committee (contact via XXX) for researchers who meet the criteria for access to confidential data.*<br><br>*The data underlying the results presented in the study are available from (include the name of the third party*	The dataset utilized for this study is accessible in the following DHS repository https://dhsprogram.<br>com/data/available-datasets.cfm.

and contact information or URL).<br>• This text is appropriate if the data are owned by a third party and authors do not have permission to share the data.<br><br>* typeset	
Additional data availability information:	

Powered by Editorial Manager® and ProduXion Manager® from Aries Systems Corporation

Manuscript
Click here to access/download;Manuscript;SANC_Manuscript_Final_2025062

Accounting for overdispersion in skilled antenatal care: Identifying determinants using Bangladesh Demographic and Health Survey 2022 data

Md. Muddasir Hossain Akib[1], Farhia Azrin[2*], Bikash Pal[1], Md. Mustain Billah[1]

[1] Department of Statistics, University of Dhaka, Dhaka, Bangladesh

[2] Institute of Statistical Research and Training, University of Dhaka, Dhaka, Bangladesh

[*] fazrin@isrt.ac.bd (FA)

Abstract

One of the major components required to ensure safe motherhood is taking a sufficient number of skilled antenatal care (SANC) visits by women during pregnancy. Various social, demographic, and economic factors have a strong influence on it. According to the World Health Organization (WHO), four or more antenatal care (ANC) visits are required for uncomplicated pregnancies and delivery of a healthy baby. SANC serves this purpose efficiently. As a result, modeling the number of SANC visits and identifying its determinants is crucial for the Bangladesh government in adopting appropriate policies and programs. In this study, we used score tests to assess dispersion in the count data and selected the Generalized Poisson Regression Model (GPRM) based on the lowest AIC (15,839.2) and BIC (16,001.7) values. Analysis of BDHS 2022 data (n = 3,839) revealed that only 38.6% of women received the recommended four or more SANC visits, with an overall mean of 3.06 visits. Key predictors of higher SANC utilization included urban residence (IRR = 1.09, $p < 0.01$), higher women's education (IRR = 1.57, $p < 0.001$), exposure to media (IRR = 1.16, $p < 0.001$), and being in the richest wealth quintile (IRR = 1.22, $p < 0.001$). In contrast, women in the Sylhet region (IRR = 0.89, $p = 0.009$) and those with higher birth order had lower SANC visit rates.

Introduction

Maternal mortality is a momentous concern in developing countries. During the pregnancy period women may ~~carry~~ some complications ~~which can lead the mothers and infants towards death~~. Globally, around 810 maternal mortality occurs every day, mostly resulting from complications during pregnancy [1]. Furthermore, these pregnancy or childbirth related complications lead to 300,000 women's deaths annually, the majority of these occurring in low-resource settings, which are largely preventable [2, 3].The highest number of deaths occurs in Southern Asia, accounting for 66%, followed by Sub-Saharan Africa

(20%), which accounts for almost 86% of the total occurrence globally [4]. It is evident that the most significant causes of death and disabilities occurring among women in Bangladesh are due to the complications of pregnancy and child delivery [5-7]. These unwanted deaths occurring in pregnancy and delivery period can be decreased with the aid of using the right care and scientific solutions provided by hospitals and clinics [8].

Antenatal care (ANC) is a set of necessary routine tests and counselling performed to train and prepare pregnant women for safe deliveries. ANC emphasizes maternal health and offers a huge platform for numerous health functions, diagnoses health problems and provides a right way to comply with the physical condition of infant and mothers. Ensuring the health of mother and infant by the end of the pregnancy is considered the ultimate goal of ANC [9]. ANC-related healthcare has been sustained globally, originating nearly a century ago from models first developed in Europe [10]. The pregnant mothers become enlightened and educated about possible pregnancy and delivery complications and their possible remedies throughout this medical supervision [11]. It is essential to take ANC-related services to lessen the risk of maternal and child deaths [12]. The World Health Organization (WHO) recommends that pregnant women take at least eight ANC examinations performed by skilled attendants to ensure safe motherhood during the whole period [13]. If a pregnant woman receives ANC-related services from professionally trained providers, then such healthcare-related services are defined as skilled ANC (SANC), as the providers elevate the required knowledge of safer motherhood-related practices and raise awareness among pregnant women to maintain healthy conditions throughout pregnancy [6]. However, according to available evidence, globally around 90% women utilize ANC by skilled providers at least one time, but only 60% of pregnant women went for skilled ANC for at least four times which is recommended by WHO [14].

Bangladesh is a developing country, and the health status of this country is gradually improving compared to the past decades. But, in the sector of maternal health this country is still lagging behind. It is evident that, the maternal mortality rate in Bangladesh has remained the same in past years with an estimated number of 196 maternal deaths per 100,000 live births which was almost similar in 2010 [15]. The target of sustainable development goals (SDGs) 3 regarding maternal mortality is to reduce the maternal death rate to less than 70 per 100,000 live births by 2030, which is a challenging issue for Bangladesh [16]. As per the report of Bangladesh Health Facility Survey 2017 (BDHS 2017), almost 99% healthcare facilities in Bangladesh offer ANC services, but only 4% are ready to provide quality ANC services [17]. Due to this, women are usually unable to receive the recommended ANC services during their pregnancy [18]. In the past, several studies conducted in Bangladesh revealed various factors to be directly associated with the antenatal healthcare of women during their pregnancy [19-22]. The available research on ANC mainly analyzed household-level data. It showed that region, type of place of residence, mother's age, mother's education, partner's education, the total number of children ever born, wealth index, and exposure to mass media have a significant impact on the pursuit of ANC-related healthcare [3, 23-25].

The number of SANC visits is count data and usually Poisson regression model (PRM) is used to handle this type of data [11, 26]. Although in real life, mostly count data are either overdispersed or underdispersed, as a result PRM cannot be applied [27]. By ignoring the dispersed nature of count data in modelling may provide misleading interpretations [28]. To the depth of authors knowledge, no study had conducted any hypothesis testing on count data of SANC services of pregnant women to check if the data is truly dispersed. As a result, this study aims to test some hypotheses to check the dispersion of SANC data. Following this, this study aims to compare different available models for handling dispersed count data and select the best-fitting model. Finally, using the latest Bangladesh Demographic and Health

Survey 2022 (BDHS 2022) data, the study ~~wants~~ to identify the most significant factors that influence skilled ANC visits among pregnant women in Bangladesh.

Materials and methods

Data and sampling design

For the purpose of analysis, in this paper, data have been extracted from the Bangladesh Demographic and Health Survey (BDHS), 2022-23. It is a nationally representative survey that is conducted as a part of the Demographic and Health Survey (DHS) program by the United States of America (USA). The data set is a retrospective survey. The information collection method is established on a two-stage stratified cluster sampling plan. At the initial stage, 675 enumeration areas were selected where EAs were selected with probability proportional to EA size. In the next and final step, 45 households per EA were systematically selected. All ever married women aged between 15-49 years who were either usual resident of the selected households or stayed in the selected households the night before the survey were eligible for the interview. After removing all the missing cases, we ended up with a dataset including the information of 3,839 respondents.

Variables

In this study, the response variable of interest is the number of skilled antenatal care (SANC) visits received by women in Bangladesh during pregnancy. An ANC healthcare service is considered skilled if a pregnant woman receives care from one or more designated professionals such as, doctors, nurses, midwives, paramedics, family welfare visitors, sub-assistant community medical officers, or community skilled birth attendants [29]. Several socio-economic and demographic variables have been considered as explanatory variables in this study. The covariates namely are region (Dhaka, Barishal, Chittagong, Khulna, Mymensingh, Rajshahi, Rangpur, Sylhet) which are administrative divisions , type of residence

(rural, urban), women's education level (no education, primary, secondary, higher), husband's education level (no education, primary, secondary, higher), women 's age in years (<20, 20-35, >35), wealth index (poor, middle, rich; derived using PCA based on ownership of household goods) , exposure to media (exposed, unexposed) , sex of household head (female, male), birth order (1, 2-3, 4 or higher), women 's religion (Muslim, others) and finally migration (yes, no). As some variables and their corresponding categories were not directly available in the survey data, we have created and reclassified them. A pregnant woman is defined as being exposed to media if she reads newspapers or magazines, listens to the radio, or watches television at least once a week [20, 21]. The variable "migration" was calculated based on respondents who had lived in their current residence for less than two years [11, 30, 31].

Models

The data were analyzed by using bivariate and multivariate statistical methods. In bivariate analysis, chi square and ANOVA F test has been utilized to check the statistical significance of categorical and count dependent variable, respectively [32, 33]. A variable was considered significant if the p-value appeared less than 0.05.

In practice, count data may show overdispersion, therefore, it is important to consider its statistical significance. Three versions of the score test statistics are available to check the statistical significance of overdispersion.

Dean and Lawless (1989) [34]:

$$z_i = \frac{((y_i - \mu_i^2) - y_i)}{\sqrt{(2\mu_i)}}.$$

Cameron and Trivedi (1990) [35]:

$$z_i = \frac{((y_i - \mu_i^2) - y_i)}{\mu_i}.$$

Winkelmann (2008) [36]:

$$z_i = \frac{((y_i - \mu_i^2) - y_i)}{2\mu_i}.$$

We initially considered four different regression models: Poisson, negative binomial, generalized Poisson and Conway-Maxwell-Poisson [37]. The Poisson regression parametric model is the benchmark for count data [38]. Count data demonstrates the number of times an occasion took place and it cannot be demonstrated by a classical linear regression due to violation of the assumptions of continuity and normally dispersed dependent variable [39]. The regression model uses the log link, and the model is linear in terms of the logarithm of the count data and assumes equidispersion. Therefore, we applied the Poisson regression model in a generalized modeling framework to analyze the number of SANC visits taken by women during pregnancy in Bangladesh [40]. The probability mass function of Poisson regression is as follows:

$$f(Y_i = y_i \mid x_i) = \frac{(e^{-\mathrm{ui}} \times \mu_i^{\mathrm{yi}})}{y_i!}; \quad y_i = 0, 1, 2, \dots, where \quad \mu_i = e^{\mathrm{xi}'\beta}.$$

Although in real life scenario, mostly the data are overdispersed, meaning the mean and variance are not equal [41]. Due to this, other options are usually considered to fit the model. Mostly, Negative binomial model is usually used to deal the situation of overdispersion, which is a gamma mixing distribution for the mean parameter [42, 43]. The response variable Y follows negative binomial (μ_i, τ) distribution with the following probability mass function:

$$f(Y_i = y_i; \mu_i, \tau) = \frac{\Gamma\left(y_i + \frac{1}{\tau}\right)}{y_i! \, \Gamma\left(\frac{1}{\tau}\right)} (1 + \tau\mu_i)^{-\frac{1}{\tau}} \left(1 + \frac{1}{\tau\mu_i}\right)^{-y_i}; y_i = 0,1,2, \ldots$$

with $E(Y_i) = \mu_i$ and $\mathrm{Var}(Y_i) = \mu_i(1 + \tau\mu_i), \tau > 0$. The extra variation due to overdispersion is $(1 + \tau\mu_i)$ that depends on the mean μ_i. Another available option for modelling count data is Generalized Poisson (GP) which is able to deal with both overdispersion and underdispersion, although the range for underdispersion is limited [44, 45].

By considering some parametric transformation, the following probability mass function is followed by GP [46].

$$f(y_i, \mu_i, \alpha) = \left(\frac{\mu_i}{1 + \alpha\mu_i}\right)^{y_i} \frac{(1 + \alpha y_i)}{y_i!} \exp\left[\frac{-\mu_i(1 + \alpha y_i)}{1 + \alpha\mu_i}\right]; y_i = 0,1,2, \ldots$$

with $E(Y_i) = \mu_i$ and $\mathrm{Var}(Y_i) = \mu_i(1 + \alpha\mu_i)^2$. If α = 0, the GP turns into Poisson, for α > 0 and α < 0, it reflects overdispersion and underdispersion respectively. Furthermore, another suitable option to model count data is Conway-Maxwell Poisson (CoMPois) which was first presented by Conway for modeling queuing system [47]. This can deal with both underdispersion and overdispersion [48, 49]. The probability mass function of CoMPois model is

$$P(Y_i = y_i | \lambda, v) = \frac{\lambda^v}{(y_i!)^v Z(\lambda, v)}, \ y = 0,1,2, \ldots, n, \text{ where } z(\lambda, v) = \sum_{s=0}^{\infty} \frac{\lambda^s}{(s!)^v}, \lambda > 1, v \geq 0$$

with $E(Y_i) = \mu_i \approx \lambda^{1/v} - \frac{v-1}{2v}$ and $\mathrm{Var}(Y_i) \approx \frac{1}{v}\lambda^{1/v}$. Here, $z(\lambda, v)$ is the normalization constant, $v > 1$ indicates underdispersion and $v < 1$ indicates overdispersion. This model is theoretically useful in many aspects, as it is a part of the family of two parameter power series distributions. Additionally, various probability mass functions can be modelled by this such as geometric distribution ($v = 0$), Poisson distribution ($v = 1$) and Bernoulli distribution ($\lambda < 1$ and $v \to \infty$). However, standard Conway-

Maxwell Poisson distribution cannot model mean directly which is a major drawback. Therefore, we considered mean-parameterized Conway-Maxwell Poisson regression to make the interpretation simple as this model allows to model the mean counts directly [50]. The model can be used by maximizing the log-likelihood $\log L(\boldsymbol{\theta}, v) = \sum_{i=1}^{n}(y_i \log\lambda\,(\mu_i, v) - v \log(y_i!) - \log z(\lambda\,(\mu_i, v), v))$ [50]. Here the link function is $E(Y_i) = \mu_i = \exp(\boldsymbol{x}_i^{\top}\boldsymbol{\beta})$, where the vector of explanatory variables are $\boldsymbol{x}_i = (x_{i1}, \dots, x_{ij}, .. x_{ip})'$ and the vector of regression coefficients are $\boldsymbol{\beta} = \left(\beta_{i1}, \dots, \beta_{ij}, .. \beta_{ip}\right)'$ and the parameters $\boldsymbol{\theta} = (\boldsymbol{\beta},\ v)'$. For the sake of this study, the mpcmp package in R is utilized to estimate the model parameters using Newton-Raphson iteration technique [51].

To choose the most suitable model among these available options Akaike Information Criteria (AIC) and Bayesian Information Criteria (BIC) is utilized. The model with the smallest AIC and BIC is considered the best fitted model [52]. For a model with p parameters and n observations, AIC and BIC can be represented as, AIC $= -2l(\widehat{\boldsymbol{\theta}}, \boldsymbol{y}) + 2p$, BIC $= -2l(\widehat{\boldsymbol{\theta}}, \boldsymbol{y}) + p\ log(n)$, where $l(\widehat{\boldsymbol{\theta}}, y)$ represents the log-likelihood. All the statistical analyses were performed using IBM SPSS Statistics version 27 and R software version 4.3.3.

Results

Table 1 represents the summary statistics of SANC visits in various socio-economic and demographic characteristics where to explore the overall scenario, frequency of 4 or more SANC visits and mean of frequency of SANC visit was considered. It is visible that, overall, only 38.6% of women receive the recommended four or more SANC with a mean of 3.06. The analysis reflected that women's educational qualification, age of women, husband's educational qualification, economic condition of family, birth order, exposure to media, religion, migration status is significantly associated with the frequency of SANC visits. Women living in Dhaka had a rate of 40.4% of SANC utilization while in Sylhet the rate was only

30.3%. Women with higher educational status were having a higher proportion (61.7%) of receiving proper SANC visits compared to women with other educational status and the scenario is similar when considering mean of SANC visits where women with higher education had a mean of 4.4. Coverage of proper SANC visits was higher (51.2%) in urban women while the rate was only 32.4% in rural women. Women aged between 20 to 35 years and more than 35 years received 40.0% and 39.0% recommended number of SANC by WHO, respectively, where only 33.9% young women utilized this service. Women who were at highest wealth index were at a higher rate (54.8%) to receive proper SANC, on the other hand, only 24.7% and 34.5% women in poor and middle wealth index received this service. Media exposure also plays a significant role where women who are exposed to media had a higher frequency (47.2%) and mean (3.5) of SANC visit. Furthermore, women who migrated reflected a higher proportion (46.2%) of at least 4 SANC visits while 37.7% non-migrated women utilized this service.

Table 1: Mean and percentage of SANC visits by the socio-economic and demographic variables with p-values

Characteristics	Number of women (%)	% of women who had ≥ 4 SANC visits	p-value[a]	Mean of frequency of SANC visits	p-value[b]
Total	3839(100.0)	38.6		3.06	
**Region**			0.051		**<0.001**
Dhaka	586(15.3)	40.4		3.37	
Barishal	422(11.0)	33.2		2.90	
Chittagong	641(16.7)	38.8		3.08	
Khulna	431(11.2)	44.3		3.45	
Mymensingh	471(12.3)	45.0		2.92	
Rajshahi	381(9.9)	40.7		3.13	
Rangpur	451(11.7)	35.5		2.89	
Sylhet	456(11.9)	30.3		2.63	
**Residence type**			**0.006**		**<0.001**
Rural	2571(67.0)	32.4		2.74	
Urban	1268(33.0)	51.2		3.70	
**Women's education level**			**<0.001**		**<0.001**

No Education	200(5.2)	23.0		1.86	
Primary	941(23.8)	24.2		2.20	
Secondary	2028(52.8)	38.7		3.09	
Higher	697(18.2)	61.7		4.44	
**Women's age at last birth**			**<0.001**		**0.004**
<20	853(22.2)	33.9		2.83	
20-35	2783(72.6)	40.0		3.12	
>35	200(5.2)	39.0		3.13	
**Husband's education level**			**<0.001**		**<0.001**
No Education	573(14.9)	26.2		2.20	
Primary	1186(30.9)	26.8		2.41	
Secondary	1341(34.9)	41.0		3.23	
Higher	739(19.2)	62.8		4.45	
**Wealth index**			**<0.001**		**<0.001**
Middle	768(20.0)	34.5		2.85	
Poor	1550(40.4)	24.7		2.21	
Rich	1521(39.6)	54.8		4.02	
**Birth order**			**<0.001**		**<0.001**
1	1348(35.1)	42.1		3.30	
2–3	2104(54.8)	38.5		3.04	
4 or higher	387(10.1)	26.9		2.31	
**Media exposure**			**<0.001**		**<0.001**
Unexposed	1685(43.9)	27.7		2.45	
Exposed	2154(56.1)	47.2		3.53	
**Religion**			**<0.001**		**<0.001**
Others	342(8.9)	47.7		3.58	
Muslim	3497(91.1)	37.7		3.01	
**Sex of household head**			0.186		**0.014**
Female	401(10.4)	41.6		3.33	
Male	3438(89.6)	38.2		3.03	
**Migration**			**0.001**		**<0.001**
No	3426(89.2)	37.7		3.00	
Yes	413(10.8)	46.2		3.54	

[a]p-values are based on the Chi-square test for testing variation among the proportions

[b] p-values are based on the Analysis of Variance (ANOVA) and F-test for testing the variation among the means.

In Table 2, three distinct dispersion tests are done to check the overdispersion of Poisson model. All of these models revealed significance, which indicates the presence of overdispersion in the SANC count data.

Table 2: Detection of overdispersion based on score tests of significance of overdispersion to SANC count data in Bangladesh

Test	Z-Score	Standard Error (SE)	p-value
**Dean & Lawless**	-1.8	2.1	<0.10
**Cameron & Trivedi**	-2.8	1.1	<0.01
**Winkelmann**	-2.8	0.5	<0.01

Table 3 represents the Akaike Information Criterion (AIC) and Bayesian Information Criterion (BIC) to assess the model performance of four different models - PRM, GPRM, NBRM, and CMPRM which are used to fit count data. The AIC values stand for 16135, 15839, 15929, and 15880, respectively, for PRM, GPRM, NBRM, and CMPRM models. Additionally, the BIC values for PRM, GPRM, NBRM, and CMPRM models are 16291, 16002, 16091, and 16043, respectively. From both AIC and BIC metric, it is visible that the GPRM model fits the data better in comparison to the other models. The minimum AIC and BIC values for GPRM concludes it as the superior model and due to this, the results produced from the GPRM model have been prioritized for further discussion in this study.

Table 3: Selection of model based on AIC and BIC for SANC count data in Bangladesh

SANC	**AIC**				**BIC**			
	PRM	GPRM	NBRM	CMPRM	PRM	GPRM	NBRM	CMPRM
	16134.8	15839.2	15928.5	15880.6	16291.1	16001.7	16091.1	16043.2

Table 4 illustrates the factors that are influencing the usage of SANC. According to the table, region, place of residence, educational qualification of women, age of women, educational qualification of husband, economical position in society, birth order, exposure to mass media, religion, sex of family head, migration

status, number of living children have a significant impact on the usage of SANC. The administrative division played a crucial role where the rate of SANC utilization was 11% lower in Sylhet compared to Dhaka. The type of place of residence plays an important role in terms of receiving proper SANC visits. The rate of SANC visit is 9% higher in urban women compared to rural women. The educational qualification of women is significantly impacting the rate of SANC visit. Women with primary, secondary, and higher education have 22%, 44% and 57% higher rate of receiving SANC, respectively, compared to women with no education. Young women are significantly less likely to receive SANC as the rate of receiving SANC is 13% and 30% higher in women aged between 20 to 35 years and more than 35 years compared to women aged less than 20 years. Women who are economically having a lower and higher condition in society have 0.86- and 1.22-times rate of receiving SANC respectively compared to middle class women. Birth order is negatively associated with the rate of receiving SANC. Having birth order of 2 or 3 and 4 or higher had 9% and 20% lower rates of receiving SANC compared to the first birth order. Exposure to the media also plays a vital role. Women exposed to mass media had a 16% higher rate compared to unexposed women. Migration is very closely associated with the rate of SANC visits where migrated women had a 7% higher rate of SANC usage compared to unmigrated women.

Table 4: Effect of the selected covariates on the SANC visits along with p-values and IRRs, obtained from the Generalized Poisson Regression Model (GPRM)

Covariate	Estimate	SE	p-value	IRR
**Region**				
Dhaka				
Barishal	-0.000	0.044	0.998	1.00
Chittagong	0.034	0.038	0.374	1.03
Khulna	0.034	0.041	0.411	1.03
Mymensingh	0.069	0.042	0.100	1.07
Rajshahi	-0.017	0.044	0.688	0.98
Rangpur	-0.050	0.044	0.258	0.95
Sylhet	-0.116	0.044	**0.009**	0.89

**Residence type**				
Rural				
Urban	0.090	0.024	**<0.001**	1.09
**Women's education level**				
No Education				
Primary	0.203	0.071	**0.004**	1.22
Secondary	0.362	0.070	**<0.001**	1.44
Higher	0.452	0.076	**<0.001**	1.57
**Women's age at last birth**				
<20				
20-35	0.120	0.033	**<0.001**	1.13
>35	0.265	0.060	**<0.001**	1.30
**Husband's education level**				
No Education				
Primary	-0.007	0.042	0.868	0.99
Secondary	0.115	0.043	**0.007**	1.12
Higher	0.267	0.049	**<0.001**	1.31
**Wealth index**				
Middle				
Poor	-0.145	0.034	**<0.001**	0.86
Rich	0.198	0.031	**<0.001**	1.22
**Birth order**				
1				
2–3	-0.097	0.028	**0.001**	0.91
4 or higher	-0.217	0.051	**<0.001**	0.80
**Media exposure**				
Unexposed				
Exposed	0.144	0.025	**<0.001**	1.16
**Religion**				
Others				
Muslim	-0.098	0.037	**0.007**	0.91
**Sex of household head**				
Female				
Male	-0.059	0.035	0.091	0.94
**Migration**				
No				
Yes	0.065	0.034	0.058	1.07

Discussion

Using the latest nationally representative dataset (BDHS 2022) from Bangladesh, the focus of this study was to examine the dispersion of SANC visit data to identify the appropriate model, as well as to determine the key factors that influence the visit. The findings reflect that the SANC data is truly dispersed in nature, as a result model that can deal the dispersion gives a better fit to this data. Additionally, predisposing factors such as age, educational qualification of women and husband were significantly associated with skilled ANC visit. Moreover, enabling factors such as place of residence, wealth status, exposure to mass media had similar association. Furthermore, this study also found that migration status also impacted the frequency of SANC.

No prior study has conducted any hypothesis testing for overdispersion on count data of SANC in Bangladesh. This study fills the gap by applying three well established statistical tests to verify the presence of dispersion in the data. Particularly from Cameron & Trivedi and Winkelmann tests, it is strongly evident that the data is overdispersed. As a result, alternative models for count data have been utilized to model the data for accurate inference. Among all other suitable models, the generalized Poisson regression model (GPRM) showed the lowest AIC and BIC values. Therefore, this study has used GPRM to model the data to find the factors that impact the utilization of SANC.

Age impacts the exposure towards utilizing SANC service. This study found that, younger women are less likely to utilize this service and this finding is in line with previous research [53]. A study conducted over East African countries investigated that, older women are aware regarding the importance of receiving skilled ANC care [54]. According to WHO, social stigma and low education limits care-seeking among younger mothers [55]. Additionally, shyness, lack of family support, misconception regarding ANC care, fear of medical procedures might be some possible reason for not seeking proper care among young mothers.

The findings of this study show that women with education are more prone to receive SANC service from skilled providers compared to mothers with no education, which is consistent with the findings of other studies from different parts of the world [24, 53, 56-60]. The possible explanation for this scenario might be the awareness and knowledge of women regarding their health which comes with education.

Wealth status is a major determining factor to receive SANC. According to the findings of the study, women in the higher wealth quintile is more likely to receive SANC, consistent with previous research findings [53, 56, 58, 60, 61]. Affordability of medical cost, easier access to medical services and knowledge about the importance of ANC service might be probable explanation for this scenario. Surprisingly, women with lower wealth index are also more likely to receive SANC compared to middle class women. Although this finding is contradictory but free health care services, community-based outreach, NGO funded clinics might be a possible reason for this scenario.

Moreover, women residing in rural areas were less likely to utilize SANC compared to urban women. This finding is consistent with former research [60, 62]. A study found that, walking long distance to reach health facilities, insufficient midwives, rude behavior of healthcare providers are contributing factor less utilization of ANC service among rural mothers [63]. Additionally, financial difficulties, cultural beliefs, lack of awareness, and less availability of quality hospitals might be some others factors to influence rural mothers in receiving less SANC.

Furthermore, this study found that exposure to media significantly impacts the uptake of SANC which is parallel with previous research [24, 53, 56, 64, 65]. Increased awareness, empowerment through information, positive health messaging may influence this pattern. They have better access to information about the importance of ANC compared to unexposed mothers and this lead them towards care-seeking behavior [65].

Migration also impacts the frequency of SANC visits. Migrated women are more likely to utilize SANC visits compared to non-migrated women. Usually, people migrate from rural to urban areas and this may have an impact on utilizing the service. The possible explanation for this scenario might be easy access to healthcare, supportive social networks and potential positive influence of surroundings.

Conclusion

This study highlights significant socio-demographic and economic determinants influencing the number of skilled antenatal care (SANC) visits among women in Bangladesh, using the most recent BDHS 2022 data. Statistical tests confirmed overdispersion in the count data, and the Generalized Poisson Regression Model provided the best fit. Key factors positively associated with higher SANC utilization include maternal education, urban residence, media exposure, wealth status, and migration. Findings underscore the need for targeted interventions—especially among rural, less-educated, and economically disadvantaged women—to improve SANC coverage and ultimately reduce maternal and neonatal health risks. We recommend expanding community-based outreach programs in underserved regions and enhancing media campaigns focused on maternal health education. Additionally, increasing the availability of trained health providers in rural areas and integrating ANC awareness into school curricula can further boost service utilization and safe motherhood outcomes.

Limitations

This study has several limitations. First, the analysis is based on cross-sectional data from the BDHS 2022, which limits the ability to establish causal relationships between predictors and SANC visits. Second, some important factors such as cultural beliefs, quality of care, and availability of health facilities could not be included due to data limitations. Third, recall bias may affect responses related to ANC visits,

especially among women reporting events from several months prior. Lastly, while the study accounts for multiple socio-demographic variables, potential unobserved confounders may still influence the results.

Acknowledgement

The authors would like to express their sincere gratitude to the Demographic and Health Surveys (DHS) Program for granting access to the BDHS 2022 dataset used in this study.

Author contribution

Conceptualization: Md. Muddasir Hossain Akib

Writing – Original Draft Preparation: Farhia Azrin, Md. Muddasir Hossain Akib

Writing – Review & Editing: Bikash Pal

Formal Analysis: Md. Mustain Billah, Md. Muddasir Hossain Akib, Farhia Azrin

References

1. Ahinkorah, B.O., et al., *Factors associated with the number and timing of antenatal care visits among married women in Cameroon: evidence from the 2018 Cameroon Demographic and Health Survey.* J Biosoc Sci, 2022. **54**(2): p. 322-332.
2. Alkema, L., et al., *Global, regional, and national levels and trends in maternal mortality between 1990 and 2015, with scenario-based projections to 2030: a systematic analysis by the UN Maternal Mortality Estimation Inter-Agency Group.* The Lancet, 2016. **387**(10017): p. 462-474.
3. Sarker, B.K., et al., *Status of the WHO recommended timing and frequency of antenatal care visits in Northern Bangladesh.* PLOS ONE, 2020. **15**(11): p. e0241185.
4. World Health, O., *Trends in maternal mortality 2000 to 2017: estimates by WHO, UNICEF, UNFPA, World Bank Group and the United Nations Population Division: executive summary*. 2019, World Health Organization: Geneva.
5. Chowdhury, R.I., et al., *Determinants of antenatal morbidity: a multivariate analysis.* World Health Popul, 2007. **9**(3): p. 9-18.
6. Jamee, A.R., K. Kumar Sen, and W. Bari, *Skilled maternal healthcare and good essential newborn care practice in rural Bangladesh: A cross-sectional study.* Health Sci Rep, 2022. **5**(5): p. e791.
7. Pal, B. and A.R. Jaamee, *Analyzing Infant Mortality in Rural Bangladesh: A Frailty Modeling Approach.* Dhaka University Journal of Science, 2021. **69**(2): p. 63-69.
8. World Health Organization. *Maternal mortality. Geneva, Switzerland*. 2022.
9. AbouZahr, C., *Safe motherhood: a brief history of the global movement 1947-2002.* Br Med Bull, 2003. **67**: p. 13-25.
10. Oakley, A., *The origins and development of antenatal care*, in *Effectiveness and satisfaction in antenatal care*. 1982, Spastics International Medical Publications/William Heinemann Medical Books: London. p. 1-21.
11. Hossain Akib, M.M. and B. Pal, *Analyzing Overdispersed Skilled Antenatal Care Visits of Pregnant Women in Bangladesh Using Generalized Poisson Regression Model.* Dhaka University Journal of Science, 2023. **71**(1): p. 69-75.
12. Pandit, R.D., *Role of Antenatal Care in Reducing Maternal Mortality.* Asia-Oceania Journal of Obstetrics and Gynaecology, 1992. **18**(1): p. 1-6.
13. World Health Organization. *New guidelines on antenatal care for a positive pregnancy experience*. 2018 November 29, 2018].
14. UNICEF, *Global databases, based on MICS, DHS and other nationally representative sources 2008-2014*. 2016.
15. MMS (Maternal Mortality Survey), *Bangladesh Maternal Mortality and Health Care Survey (2016)*. 2017: Dhaka, Bangladesh; Chapel Hill, NC, USA.
16. World Health Organization. *Sustainable Development Goals (SDG) 3. Ensure healthy lives and promote wellbeing for all at all ages*. 2018.
17. Hakim, S., et al., *Are Bangladeshi healthcare facilities prepared to provide antenatal care services? Evidence from two nationally representative surveys.* PLOS Global Public Health, 2022. **2**(7): p. e0000164.
18. Jo, Y., et al., *Antenatal care in rural Bangladesh: current state of costs, content and recommendations for effective service delivery.* BMC Health Services Research, 2019. **19**(1): p. 861.

19. Chowdhury, R.I., et al., *Delivery complications and healthcare-seeking behaviour: the Bangladesh Demographic Health Survey, 1999-2000.* Health Soc Care Community, 2007. **15**(3): p. 254-64.
20. Koenig, M.A., et al., *Maternal health and care-seeking behavior in Bangladesh: findings from a national survey.* Int Fam Plan Perspect, 2007. **33**(2): p. 75-82.
21. Amin, R., N.M. Shah, and S. Becker, *Socioeconomic factors differentiating maternal and child health-seeking behavior in rural Bangladesh: A cross-sectional analysis.* Int J Equity Health, 2010. **9**: p. 9.
22. Islam, M.M. and M.S. Masud, *Health care seeking behaviour during pregnancy, delivery and the postnatal period in Bangladesh: Assessing the compliance with WHO recommendations.* Midwifery, 2018. **63**: p. 8-16.
23. Rahman, A., et al., *Trends, determinants and inequities of 4(+) ANC utilisation in Bangladesh.* J Health Popul Nutr, 2017. **36**(1): p. 2.
24. Ali, N., et al., *Predictors of Optimal Antenatal Care Service Utilization Among Adolescents and Adult Women in Bangladesh.* Health Serv Res Manag Epidemiol, 2018. **5**: p. 2333392818781729.
25. Pervin, J., et al., *Determinants of utilization of antenatal and delivery care at the community level in rural Bangladesh.* PLoS One, 2021. **16**(9): p. e0257782.
26. Hossain, Z., et al., *Modelling zero-truncated overdispersed antenatal health care count data of women in Bangladesh.* PloS one, 2020. **15**(1): p. e0227824.
27. Zeileis, A., C. Kleiber, and S. Jackman, *Regression Models for Count Data in R.* Journal of Statistical Software, 2008. **27**(8): p. 1 - 25.
28. Hossain, Z. and Maria, *Analyzing Overdispersed Antenatal Care Count Data in Bangladesh: Mixed Poisson Regression with Individual-Level Random Effects.* Austrian Journal of Statistics, 2021. **50**(4): p. 78-90.
29. Akib, M.M.H., F. Afroz, and B. Pal, *Beyond averages: dissecting urban-rural disparities in skilled antenatal care utilization in Bangladesh - a conway-maxwell-poisson regression analysis.* BMC Pregnancy Childbirth, 2025. **25**(1): p. 119.
30. National Institute of Population Research and Training (NIPORT), Mitra and Associates, and I. International, *Bangladesh Demographic and Health Survey 2017–18*. 2017: Dhaka, Bangladesh and Rockville, Maryland, USA.
31. Akib, M.M.H. and B. Pal, *Analyzing Number of Children Ever Born in Bangladesh Using Generalized Poisson Regression Model.* Dhaka University Journal of Science, 2024. **72**(2): p. 1-6.
32. Amin, M.T., et al., *Prevalence and correlates of anxiety and depression among ever-married reproductive-aged women in Bangladesh: national-level insights from the 2022 Bangladesh Demographic and Health Survey.* BMC Public Health, 2025. **25**(1): p. 1143.
33. Akter, S., et al., *Rise of Women: A Decadal Journey of Women's Empowerment in Bangladesh (2010-2020): Rise of Women: A Decadal Journey of Women's Empowerment.* Dhaka University Journal of Science, 2024. **72**(2): p. 34-44.
34. Dean, C. and J.F. and Lawless, *Tests for Detecting Overdispersion in Poisson Regression Models.* Journal of the American Statistical Association, 1989. **84**(406): p. 467-472.
35. Cameron, A.C. and P.K. Trivedi, *Regression-based tests for overdispersion in the Poisson model.* Journal of Econometrics, 1990. **46**(3): p. 347-364.
36. Winkelmann, R., *Econometric Analysis of Count Data*. 2008.
37. McCullagh, P., *Generalized Linear Models*. 2019.
38. Bhowmik, K.R., S. Das, and M.A. Islam, *Modelling the number of antenatal care visits in Bangladesh to determine the risk factors for reduced antenatal care attendance.* PLoS One, 2020. **15**(1): p. e0228215.

39. Hayat, M.J. and M. Higgins, *Understanding poisson regression.* J Nurs Educ, 2014. **53**(4): p. 207-15.
40. Shahjahan, M., et al., *Factors associated with use of antenatal care services in a rural area of Bangladesh.* South East Asia Journal of Public Health, 2013. **2**(2): p. 61-66.
41. Afroz, F., et al., *Impact of parental education on number of under five children death per mother in Bangladesh.* PLOS ONE, 2025. **20**(2): p. e0318787.
42. Chatfield, C., A.S. Ehrenberg, and G.J. Goodhardt, *Progress on a simplified model of stationary purchasing behaviour.* Journal of the Royal Statistical Society: Series A (General), 1966. **129**(3): p. 317-367.
43. Manton, K.G., M.A. Woodbury, and E. Stallard, *A variance components approach to categorical data models with heterogeneous cell populations: analysis of spatial gradients in lung cancer mortality rates in North Carolina counties.* Biometrics, 1981: p. 259-269.
44. Consul, P.C. and F. Famoye, *Generalized Poisson regression model.* Communications in Statistics - Theory and Methods, 1992. **21**(1): p. 89-109.
45. Hayati, M., K. Sadik, and A. Kurnia, *Conwey-Maxwell Poisson Distribution: Approach for Over-and-Under-Dispersed Count Data Modelling.* IOP Conference Series Earth and Environmental Science, 2018. **187**(1): p. 012039.
46. Famoye, F., *Restricted generalized Poisson regression model.* Communications in Statistics-theory and Methods - COMMUN STATIST-THEOR METHOD, 1993. **22**: p. 1335-1354.
47. Conway, R.I., *A queueing model with state dependent service rate.* Journal of Industrial Engineering, 1961. **12**: p. 132.
48. Shmueli, G., et al., *A useful distribution for fitting discrete data: revival of the Conway–Maxwell–Poisson distribution.* Journal of the Royal Statistical Society: Series C (Applied Statistics), 2005. **54**(1): p. 127-142.
49. Lord, D., S.D. Guikema, and S.R. Geedipally, *Application of the Conway-Maxwell-Poisson generalized linear model for analyzing motor vehicle crashes.* Accid Anal Prev, 2008. **40**(3): p. 1123-34.
50. Huang, A., *Mean-parametrized Conway–Maxwell–Poisson regression models for dispersed counts.* Statistical Modelling, 2017. **17**(6): p. 359-380.
51. Fung, H.T., et al., *mpcmp: Mean-parametrized Conway-Maxwell Poisson Regression.*
52. Akaike, H., *Information Theory and an Extension of the Maximum Likelihood Principle*, in *Selected Papers of Hirotugu Akaike*, E. Parzen, K. Tanabe, and G. Kitagawa, Editors. 1998, Springer New York: New York, NY. p. 199-213.
53. Akter, E., et al., *Levels and determinants of quality antenatal care in Bangladesh: Evidence from the Bangladesh Demographic and Health Survey.* PLOS ONE, 2023. **18**(5): p. e0269767.
54. Tessema, Z.T. and A. Minyihun, *Utilization and Determinants of Antenatal Care Visits in East African Countries: A Multicountry Analysis of Demographic and Health Surveys.* Advances in Public Health, 2021. **2021**(1): p. 6623009.
55. World Health, O., *Adolescent pregnancy: adolescence is a time of opportunity during which a range of actions can be taken to set the stage for healthy adulthood: factsheet*. 2014, World Health Organization: Geneva.
56. Islam, M.M. and M.S. Masud, *Determinants of frequency and contents of antenatal care visits in Bangladesh: Assessing the extent of compliance with the WHO recommendations.* PLOS ONE, 2018. **13**(9): p. e0204752.
57. Joshi, C., et al., *Factors associated with the use and quality of antenatal care in Nepal: a population-based study using the demographic and health survey data.* BMC Pregnancy Childbirth, 2014. **14**: p. 94.

58. Fagbamigbe, A.F. and E.S. Idemudia, *Assessment of quality of antenatal care services in Nigeria: evidence from a population-based survey.* Reprod Health, 2015. **12**: p. 88.
59. Kabir, M., et al., *Determinants of utilization of antenatal care services in Kumbotso Village, northern Nigeria.* Trop Doct, 2005. **35**(2): p. 110-1.
60. Dickson, K.S., et al., *Determinants of choice of skilled antenatal care service providers in Ghana: analysis of demographic and health survey.* Maternal Health, Neonatology and Perinatology, 2018. **4**(1): p. 14.
61. Singh, L., et al., *Measuring quality of antenatal care: a secondary analysis of national survey data from India.* Bjog, 2019. **126 Suppl 4**: p. 7-13.
62. Dickson, K.S., E.K. Darteh, and A. Kumi-Kyereme, *Providers of antenatal care services in Ghana: evidence from Ghana demographic and health surveys 1988-2014.* BMC Health Serv Res, 2017. **17**(1): p. 203.
63. Kambala, C., et al., *Barriers to maternal health service use in Chikhwawa, Southern Malawi.* Malawi Med J, 2011. **23**(1): p. 1-5.
64. Acharya, D., et al., *Impact of mass media on the utilization of antenatal care services among women of rural community in Nepal.* BMC Res Notes, 2015. **8**: p. 345.
65. Edward, B., *Factors influencing the utilisation of antenatal care content in Uganda.* Australas Med J, 2011. **4**(9): p. 516-26.
