# Supplementary material for: Accounting for overdispersion in skilled antenatal care: Identifying determinants using Bangladesh Demographic and Health Survey 2022 data
Source: PLoS One. 2026 Apr 8;21(4):e0346258. doi: 10.1371/journal.pone.0346258 (PMC13061173; doi:10.1371/journal.pone.0346258)
Supplement: Attachment — Submitted filename: PLOS RECOMMENDATIONS.docx [file pone.0346258.s002.docx]

**TITLE: ACCOUNTING FOR OVERDISPERSION IN SKILLED ANTENATAL CARE: IDENTIFYING DETERMINANTS USING BANGLADESH DEMOGRAPHIC AND HEALTH SURVEY 2022 DATA**

**PRESENTATION OF THE RESULTS**

1. **To Validate Observations Statistically**:
   - Your summary shows differences in SANC (Skilled Antenatal Care) visits across education, location, age, and wealth index.
   - Statistical tests (e.g., chi-square, ANOVA, regression) help determine if these differences are **statistically significant** or just due to chance.
2. **To Identify Key Predictors**:
   - Understanding which factors (education, wealth, age, etc.) most strongly influence proper SANC visits can guide **policy and intervention**.
3. **To Control for Confounding**:
   - For example, urban women may also be more educated. A multivariate analysis (like logistic regression) helps isolate the **independent effect** of each variable.
4. **To Improve Clarity and Credibility**:
   - Presenting statistical relationships (with p-values, confidence intervals, etc.) strengthens the **scientific rigor** of your findings.

**DISCUSSION OF RESULTS**

**Please improve the discussion by the following aspects**

**Statistical Support:**

Include whether the differences in SANC uptake across wealth groups are statistically significant.

Mention any tests used (e.g., chi-square, logistic regression) and their results (e.g., p-values, odds ratios).

**Clarify the Middle-Income Paradox:**

Consider exploring why middle-income women might be underserved:

Are they excluded from free services?

Do they face barriers like cost or distance without the support that poorer women get?

**Use Data to Strengthen Claims:**

Refer to actual percentages or mean values from your study to support each point.

For example: “Women in the highest wealth quintile had a 54.8% SANC coverage, compared to 24.7% in the poorest and 34.5% in the middle-income group.”

**Policy Implications:**

Suggest what this means for health policy: Should outreach be expanded to middle-income women? Should subsidies be adjusted?
