# Supplementary material for: Accounting for overdispersion in skilled antenatal care: Identifying determinants using Bangladesh Demographic and Health Survey 2022 data
Source: PLoS One. 2026 Apr 8;21(4):e0346258. doi: 10.1371/journal.pone.0346258 (PMC13061173; doi:10.1371/journal.pone.0346258)
Supplement: Attachment — Submitted filename: Response_to_Reviewers_auresp_2.docx [file pone.0346258.s007.docx]

**Authors’ responses to the points raised by the Academic Editor and reviewers**

First of all, we would like to thank the Academic Editor and the reviewers for reviewing our joint work entitled “Accounting for overdispersion in skilled antenatal care: Identifying determinants using Bangladesh Demographic and Health Survey 2022 data” and providing their valuable suggestions about our manuscript. All the suggestions raised by the Academic Editor and reviewers have been addressed in the revised version which are categorically pointed out in the followings.

**Responses to Reviewer 2:**

The author addressed some of the comments, while other comments were not addressed. The highlighted text in the manuscript was not addressed. Can the comments be addressed, or reasons for not addressing them be provided?

**Response:** We have addressed all the reviewer comments to the best of our ability, and where certain comments could not be addressed, we have provided explanations in the manuscript and the response document. Regarding the highlighted text mentioned, we carefully checked the manuscript but did not find any highlighted sections.
